# LncRNA IMFlnc1 promotes porcine intramuscular adipocyte adipogenesis by sponging miR-199a-5p to up-regulate CAV-1

**DOI:** 10.1186/s12860-020-00324-8

**Published:** 2020-11-04

**Authors:** Jing Wang, Ming-yue Chen, Jun-feng Chen, Qiao-ling Ren, Jia-qing Zhang, Hai Cao, Bao-song Xing, Chuan-ying Pan

**Affiliations:** 1 Henan Key Laboratory of Farm Animal Breeding and Nutritional Regulation, Institute of Animal Husbandry and Veterinary Science, Henan Academy of Agricultural Sciences, Number 116, Hua Yuan Road, Jinshui District, Zhengzhou, 450002 China; 2 Key Laboratory of Animal Genetics, Breeding and Reproduction of Shaanxi Province, Key Laboratory of Animal Biotechnology, College of Animal Science and Technology, Northwest A&F University, Ministry of Agriculture, Number 22, Xi Nong Road, Yangling, 712100 Shaanxi China; 3 Henan Xing Rui Agriculture and Animal Husbandry Technology Co., LTD, Number 59, Jie Fang Road, Xinxian, Xinyang, 465550 China

**Keywords:** lncRNA, ceRNA, Intramuscular adipocyte adipogenesis, miR-199a-5p, CAV-1

## Abstract

**Background:**

Local Chinese local pig breeds have thinner muscle fiber and higher intramuscular-fat (IMF) content. But its regulation mechanism has not been discussed in-depth. Studies indicated that long non coding RNAs (lncRNAs) play important role in muscle and fat development.

**Results:**

The lncRNAs expressional differences in the longissimus dorsi (LD) muscle were identified between Huainan pigs (local Chinese pigs, fat-type, HN) and Large White pigs (lean-type, LW) at 38, 58, and 78 days post conception (dpc). In total, 2131 novel lncRNAs were identified in 18 samples, and 291, 305, and 683 differentially expressed lncRNAs (DELs) were found between these two breeds at three stages, respectively. The mRNAs that co-expressed with these DELs were used for GO and KEGG analysis, and the results showed that muscle development and energy metabolism were more active at 58 dpc in HN, but at 78 dpc in LW pigs. Muscle cell differentiation and myofibril assembly might associated with earlier myogenesis and primary-muscle-fiber assembly in HN, and cell proliferation, insulin, and the MAPK pathway might be contribute to longer proliferation and elevated energy metabolism in LW pigs at 78 dpc. The PI3K/Akt and cAMP pathways were associated with higher IMF deposition in HN. Intramuscular fat deposition-associated long noncoding RNA 1 (IMFlnc1) was selected for functional verification, and results indicated that it regulated the expressional level of caveolin-1 (CAV-1) by acting as competing endogenous RNA (ceRNA) to sponge miR-199a-5p.

**Conclusions:**

Our data contributed to understanding the role of lncRNAs in porcine-muscle development and IMF deposition, and provided valuable information for improving pig-meat quality.

**Supplementary Information:**

The online version contains supplementary material available at 10.1186/s12860-020-00324-8.

## Background

In daily human diet, the main source of animal protein is pork, especially in China, where pork is consumed the most by residents. Recently, more and more attention has been paid to meat quality. The diameter of muscle fibers, the content of intramuscular fat, and muscle fiber types are all involved in regulating meat-quality traits [1, 2]. Meat-quality traits are also regulated by nutrition, feeding conditions, stress, and, most importantly, by genetic influence [3]. Lean-type pigs like Large White (LW), Landrace (LR), and Duroc generally have advantages such as high meat production, a fast growth rate, and a low feed-to-meat ratio. Most local Chinese pig breeds are fat-type, and they generally have bright red meat, thin muscle fibers, high intramuscular fat content, and more glycolytic muscle fibers. Two pig types with differences in meat-quality traits were a potentially good model to study the genetic-regulation mechanism of potential muscle-phenotype differences [4].

Some studies focused on the differences between lean and fat-type pigs recently. The transcriptome of the longissimus dorsi (LD) muscle between German Landrace (more obese) and Pietrain (more leaner) fetuses at embryonic (35, 63, and 91 days post conception (dpc)) and adult period (180 days postnatal) was compared, and results indicated that the differential expressed genes were related with muscle development stages and meat quality [5]. Xu et al. compared the transcriptome of LD muscle between Northeast Min (fat-type) and Changbaishan (lean-type) wild boar at the age of 42 days; the difference between these two breeds was mainly attributed to development of myofiber, and metabolism of lipid [6]. The LD muscle miRNAomes between Meishan (MS) and LW pigs at 35 dpc were compared, and results revealed that, in 87 differentially expressed miRNAs mainly enriched in mammalian target of rapamycin (mTOR), muscle contraction, wingless/integrated (WNT), and mitogen-activated protein kinase (MAPK) signaling pathway, and the highly abundant miRNAs in these two pigs were different [7]. At postnatal day 240, the miRNAs with different expressional level in the LD muscle of LR and Tongcheng (TC) pigs were mainly enriched in the muscle-organ development and oxidative stress [8]. Sun et al., compared the transcriptome of LD muscle between LR and Lantang (LT) pigs at the age of 7 days. They found 547 DEGs, 5566 differentially expressed lncRNAs (DELs), and 4360 differentially expressed circRNAs (DECs), respectively [9]. DNA methylation in TC, LR and Wuzhishan (WZS) pigs’ LD muscle at the age of 240 days were compared, muscle development and lipid metabolism were enriched for the differently methylated genes [10]. Meat quality differences between fat- and lean-type pigs were mainly due to muscle development and lipid metabolism.

In previous studies, LD muscle samples were collected from fetal, new-born, or adult pigs. Zhao et al., identified the characterization of large intergenic noncoding RNAs (lincRNAs) in the LD muscle of TC pigs at 50, 55, 60, 65, and 75 dpc [11], but the expressional differences of lncRNAs during the embryonic stage between fat- and lean-type have not been systematic studied. During the embryonic stage, there are two major fiber generation periods. The first wave is myoblasts differentiating into primary myofibers (35—60 dpc), and the second is the secondary myofibers forming a base on primary myofibers (54—90 dpc) [12]. Before birth, the total number of fibers (TNF) is fixed, for postnatal, the development of muscle is mainly growth and maturation, so the embryonic stage is vital in skeletal muscle development [13]. Researches also showed that adipogenic differentiation and the second wave of fiber generation are simultaneous in cattle. In the later stages of embryonic development, the differences in intramuscular fat between TC and Yorkshire (YK) pigs were significantly [12]. In production, increasing the nutritional level of females in later pregnancy could promote muscle fat content of the young animals. Therefore, the embryonic stage is a key stage for studying meat quality traits.

Recently researches indicated that long non coding RNA (lncRNA) also participated in the regulation of muscle development and lipid metabolism by competing endogenous RNA (ceRNA) mechanism. lncRNA maternally expressed gene 3 (Meg3) induced 3 T3-L1 preadipocytes adipogenesis via miR-217-Dickkopf-3 pathway [14]. In human adipose tissue-derived mesenchymal stem cells (ADSCs), Terminal differentiation-induced ncRNA (TINCR) modulated the adipogenic differentiation by forming a feedback loop with miR-31 and C/EBP-α [15]. A novel lncRNA (muscle anabolic regulator 1, MAR1) promoted muscle differentiation and regeneration by sponging miR-487b and regulating Wnt5a [16]. lncRNA TUG1 promoted VSMCs proliferation and migration via miR-145-5p-FGF10 pathway [17].

As an excellent local breed in China, Huainan (HN) pigs are mainly distributed in the upper reaches of the Huai River, showing heat- and roughage-resistance, and high intramuscular fat deposition. Huainan breed was name “fine livestock and poultry breeds in Henan province” in 1986 [18]. Previously, we studied the characterization of lncRNA in adult porcine subcutaneous fat and LD muscle [19–21]. So far, the effect of lncRNAs on the muscle development in the embryo stage of Huainan pigs hasn’t been researched. In this study, considering the temporality of myogenic differentiation, lncRNA expression profiles in LD muscle between HN pigs and LW pigs at 38, 58, and 78 dpc was compared. Considering the expressional level (FPKM > 10), transcript length (< 2000 bp), and miR-199a-5p binding site, lnc_000167 was selected from the 37 shared DELs in different stages, and it was named intramuscular fat deposition-associated long noncoding RNA 1 (IMFlnc1). Results were helpful to analyze the lncRNAs’ regulation mechanism on differences of meat quality between fat- and lean-type pigs during the embryonic period, and provide basic materials for improving meat quality traits for breeding.

## Results

### lncRNA identification in porcine LD muscle

To verify the effect of lncRNAs in LD muscle development during porcine embryonic period, we examined the DELs of LD muscles between HN and LW pigs at the three embryonic-development stages. In total, there were 1.94 billion clean reads in these 18 samples, and 79.63% mapped to the porcine reference genome (Sscrofa10.2), 12.87% were multiply mapped, 66.76% were uniquely mapped, 33.34% mapped to “+”, 33.47% mapped to “–”, 19.24% were splice reads, and 47.52% was nonsplice reads (Additional file 1: Table S1). Expression correlation between the three samples in the same treatment was from 0.953 to 0.969 (Additional file 5: Figure S1), indicating that sample selection was reasonable, and experiment results were reliable.

### lncRNA properties in porcine LD muscle

**Fig. 1:**
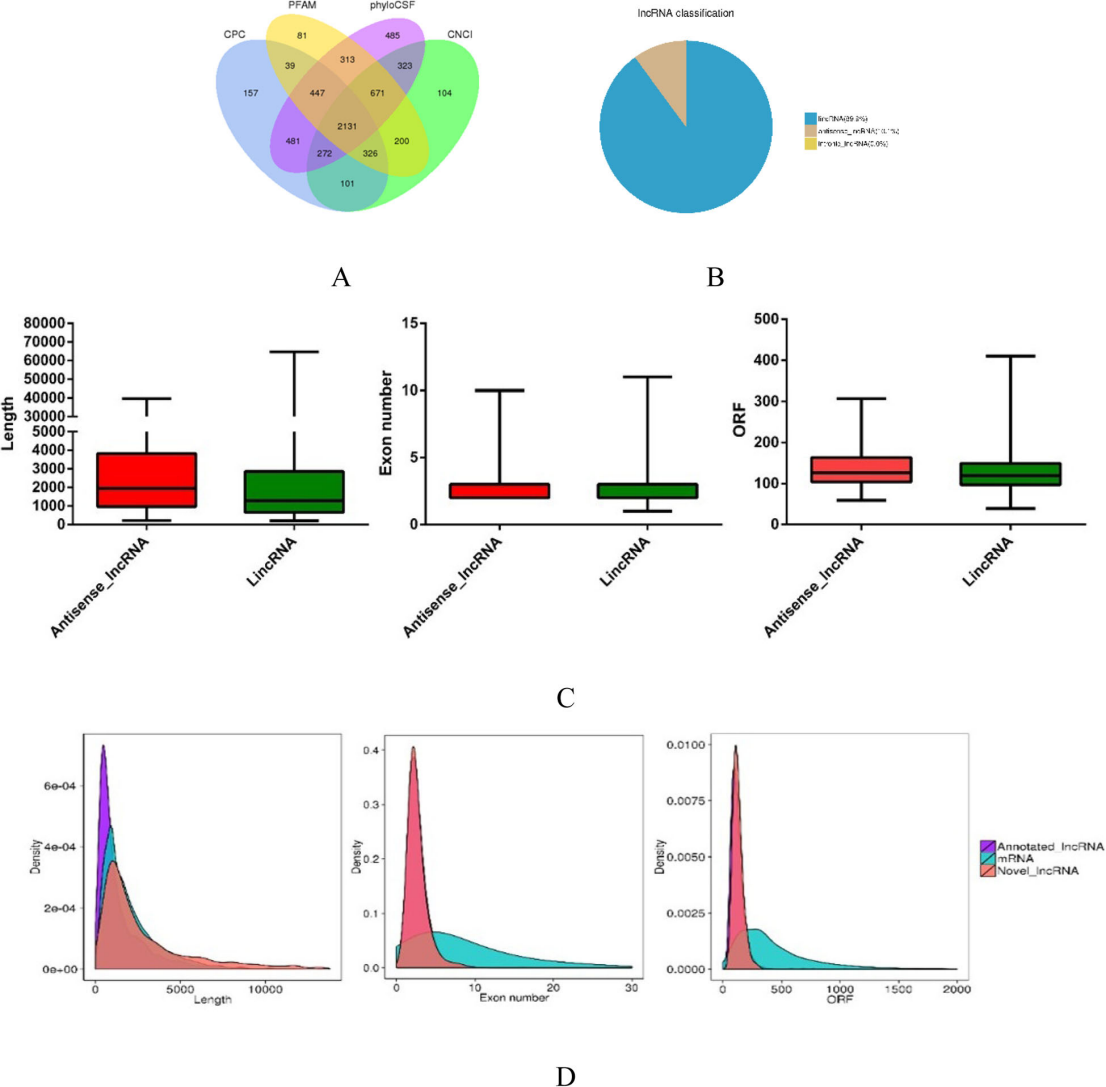
Genomic features of the identified lncRNAs in longissimus dorsi muscle tissue of HN and LW pigs at 35, 55 and 75 days post-conception. **a** Screening of candidate lncRNAs by analyzing their coding potential via CPC, CNCI, phyloCSF and PFAM. **b** The classification of the 2131 novel identified lncRNAs. **c** Length, exon number and ORF length distribution of annotated lncRNAs, novel lncRNAs and mRNAs. **d** Length, exon number and ORF length distribution of novel discovered antisense lncRNAs and lincRNAs

In total, 2131 novel lncRNAs and 2057 annotated lncRNAs were detected from 18 LD muscle samples (Fig. 1a), and only lincRNA (89.9%)and intronic lncRNA were found in the novel lncRNAs (Fig. 1b). The annotated lncRNAs’ average length was shorter than the novel lncRNAs’ average length, but there was no significant difference in open reading frame (ORF) length and exon number (Fig. 1c). For the novel lncRNAs, antisense lncRNAs’ average length was longer than lincRNAs’ average length (Fig. 1d). Information on the novel lncRNAs is shown in Additional file 2: Table S2.

### Expression difference of lncRNAs between HN and LW pigs

**Fig. 2:**
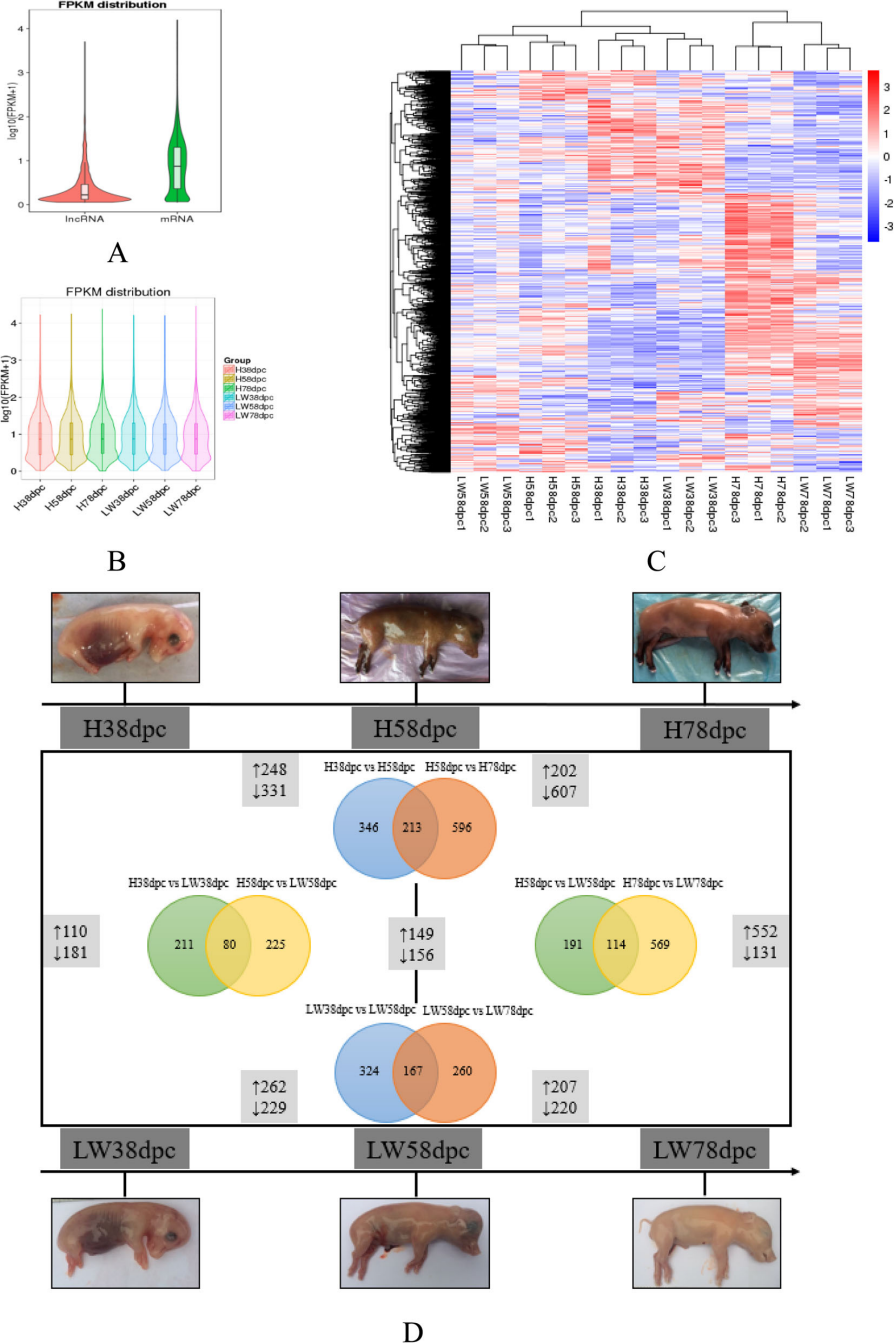
The differentially expressed lncRNAs in longissimus dorsi muscle tissue between Huainan (HN) and Large white (LW) at 38, 58 and 78 post-conception. **a** The FPKM distribution of lncRNAs and mRNAs. **b** FPKM distribution of lncRNAs in HN and LW. **c** Hierarchical clustering of differentially expressed lncRNAs. **d** The numbers of DELs between different stages in HN or LW were depicted on vertical lines. The numbers of DELs between HN and LW at each stage were depicted on horizontal lines. ↑: up-regulated, ↓: down-regulated

The expressional level of lncRNAs were lower than that of mRNAs (Fig. 2a), and lncRNAs FPKM distribution of LW pigs at 78 dpc was higher than that of the others (Fig. 2b). Systematic cluster analysis was carried out to analyze the 18 LD muscle libraries’ relationship, and results indicated that three replicates of the same sample were very conservative (Fig. 2c). The lncRNAs expression differences between the different stages were larger than those of the two breeds. There were 1155 and 751 DELs during muscle development in HN and LW pigs, respectively. In HN pigs, 579 and 809 DELs were identified at 58 vs 38 dpc and 78 vs 58 dpc, and 213 DELs were shared in these two comparisons. In LW pigs, there were 579 and 809 DELs at 58 vs 38 dpc and 78 vs 58 dpc, respectively, and 167 DELs were shared. At 78 vs 58 dpc, there were more DELs in HN than in LW pigs. There were 291, 305, and 683 DELs between these two breeds at 38, 58, and 78 dpc, and 37 DELs were shared by these three stages (Fig. 2d and Additional file 6: Figure S2 and Additional file 3: Table S3).

During embryonic muscle development, the GO enrichment of DEL-co-expressed genes in HN showed that muscle tissue development and the response to oxygen-containing compound were upregulated in 58 dpc. In LW pigs, only the response to the oxygen-containing compound was upregulated at 58 dpc, and muscle cell differentiation, myofibril assembly were only upregulated at 78 dpc; other muscle development-related pathways were continuously upregulated in both breeds. At 58 vs 38 dpc, muscle cell differentiation and myofibril assembly were only upregulated in HN at 58 dpc. Skeletal muscle tissue development was only upregulated in LW at 78 dpc (Table 1 and Additional file 3: Table S3).

**Table 1:** The differently expressed lncRNAs between different libraries

Signaling pathway	H58/H38	LW58/LW38	H78/H58	LW78/LW58	H38/LW38	H58/LW58	H78/LW78
Insulin	↑	↑	↑	↑	↓	↓	↓
Calcium	↑	↑	↑	↑	↓	↓	↓
cAMP	↑	↑		↑	↓	↓	↓
HIF-1	↑	↑	↑	↑	↓	↑	↓
Fatty acid elongation	↑	↑	↑	↑	↓		
Glycolysis/gluconeogenesis	↑	↑	↑	↑			↓
Glyoxylate and dicarboxylated metabolism	↑	↑		↑			↓
MAPK	↑	↑				↓	↓
Fatty acid degradation	↑		↑	↑	↓		↓
Biosynthesis of unsaturated fatty acid	↓	↓	↓	↓	↑↓	↓	
Metabolic pathways	↓	↓		↓			
Cardiac muscle contraction	↑	↑	↑	↑		↑↓	↓
cGMP-PKG	↑	↑	↑	↑	↓	↑↓	
Type II diabetes mellitus	↑↓	↑↓	↑	↑	↓	↓	
HCM	↑↓	↑↓	↑	↑	↑↓	↑↓	↓
ARVC	↑↓	↑↓	↑	↑↓	↑↓	↓	↑↓
Biosynthesis of amino acids	↑↓	↑	↑	↑	↑↓	↑	↓
Insulin secretion	↑	↑↓	↑	↑	↓	↓	↑
Adipocytokine pathway	↑	↓	↑	↑↓	↓	↑	↓
Focal adhesion	↑↓	↑↓	↑↓	↑↓	↑↓	↑↓	↑↓
PI3K-Akt	↑↓	↑↓	↑↓	↑↓	↑↓	↑↓	↑
ECM-receptor interaction	↑↓	↑↓	↑↓	↑↓	↑↓	↑↓	↑
Steroid biosynthesis	↑↓	↑↓	↑↓	↑↓	↑↓	↑	↑
AMPK	↑↓	↑↓	↑↓	↑↓	↓	↓	↓
Fatty acid metabolism	↑↓	↑↓		↑↓	↑↓	↑	↓
Ala, asp and glu metabolism	↑↓	↑↓	↑		↑		↑↓
Arginine and proline metabolism	↑↓	↑↓		↑↓			↑↓
Carbon metabolism	↑↓	↑↓	↑	↑↓			↓
Fructose and mannose metabolism			↑	↑		↑	↓
Rap1	↑↓	↑↓	↑			↓	↑
Protein digestion and absorption				↑		↑	↑

*Ala, asp and glu metabolism* Alanine, aspartate and glutamate metabolism, ↑ up-regulated, ↓ down-regulated, ↑↓ up-regulated and down-regulated

KEGG enrichment of DEL-co-expressed genes shown that the cyclic adenosine monophosphate (cAMP), glyoxylate and dicarboxylated metabolism, metabolic pathways were only upregulated in HN pigs at 58 dpc. Fatty acid degradation and fructose and mannose metabolism was only upregulated in LW pigs at 78 dpc. The adipocytokine pathway was upregulated in HN but downregulated in LW pigs at 58 dpc. The MAPK pathway was upregulated only at 58 dpc in both breeds, and fructose and mannose metabolism was upregulated only at 78 dpc in both breeds. DEL-co-expressed genes related to fatty acid degradation, cAMP, glyoxylate and dicarboxylated metabolism, fatty acid metabolism, metabolic pathways continuously changed only in HN or LW pigs. Genes associated with metabolism of alanine, aspartate and glutamate, and Rap1 with higher expressional level in HN pigs at 78 dpc, while genes in protein digestion and absorption with a higher level in LW pigs at 78 dpc (Table 2 and Additional file 7: Figure S3).

**Table 2:** The muscle-related GO terms of DELs co-expressed genes during muscle development stages in Huainan and Large white pigs

GO terms	H58/H38	LW58/LW38	H78/H58	LW78/LW58	H58/LW58	H78/LW78
muscle cell differentiation	↑ 9 (0.260)		↑ 9 (0.241)	↑10 (0.062)	↑ 6 (0.438)	↓ 6 (0.868)
contractile fiber	↑ 8 (0.065)	↑ 7 (0.180)	↑ 8 (0.084)	↑ 8 (0.011)	↑ 5 (0.361)	
myofibril	↑ 7 (0.089)	↑ 6 (0.268)	↑ 7 (0.106)	↑ 7 (0.027)	↑ 4 (0.415)	
regulation of glucose metabolic process	↑ 5 (0.124)	↑ 5 (0.227)	↑ 5 (0.163)	↑ 5 (0.046)	↑ 3 (0.480)	
muscle structure development	↑12 (0.157)	↑11 (0.278)	↑12 (0.174)	↑13 (0.029)	↑ 8 (0.385)	
muscle system process	↑ 9 (0.160)	↑ 8 (0.278)	↑ 6 (0.247)	↑ 9 (0.061)	↑ 6 (0.385)	
muscle tissue development	↑11 (0.148)	↑10 (0.278)	↑10 (0.212)	↑13 (0.011)		↓ 7 (0.757)
skeletal muscle tissue development	↑ 8 (0.160)	↑ 8 (0.240)		↑10 (0.011)	↑ 5 (0.415)	↓ 5 (0.778)
skeletal muscle organ development	↑ 8 (0.161)	↑ 8 (0.260)		↑10 (0.011)	↑ 5 (0.438)	↓ 5 (0.847)
muscle organ development	↑10 (0.180)	↑ 9 (0.312)		↑12 (0.013)	↑ 6 (0.480)	↓ 7 (0.608)
myofibril assembly	↑ 4 (0.124)		↑ 4 (0.163)	↑ 4 (0.046)		↓ 3 (0.544)
striated muscle tissue development	↑10 (0.180)	↑ 9 (0.334)		↑12 (0.015)		↓ 7 (0.608)
response to oxygen-containing compound	↑19 (0.124)	↑19 (0.179)				
cell proliferation				↑20 (0.195)		↓16 (0.544)

Note: ↑: up-regulated; ↓: down-regulated; numbers next to the arrow showed the number of differently expressed genes in this GO term; numbers in the brackets showed the corrected *P*-values

The results of GO enrichment of DELs between HN and LW pigs at the same stage showed that no pathway was enriched between these two breeds at 38 dpc. At 58 dpc, genes in muscle cell differentiation and eight other pathways had a lower expression level in LW pigs. At 78 dpc, the genes in seven pathways were upregulated in LW pigs. Genes associated with muscle cell differentiation, skeletal muscle tissue/organ development, muscle organ development pathways showed, higher expressional level at 58 dpc, but a lower expression level in HN pigs than that of in LW pigs (Table 1 and Additional file 3: Table S3).

KEGG analysis indicated that DEL-co-expressed genes involved in insulin, calcium, and the cAMP signaling pathway were continuously highly expressed in HN pigs. Genes in steroid biosynthesis showed a higher level in HN pigs at 58 and 78 dpc. Compared with LW pigs, genes in hypoxia-inducible factor 1 (HIF-1) and adipocytokine signaling pathway were upregulated in LW pigs at 38 and 78 dpc, but downregulated at 58 dpc. Genes involved in insulin secretion and Rap1 were downregulated at 58 dpc and upregulated at 78 dpc in HN pigs. The expression trends of biosynthesis of amino acids, fructose and mannose metabolism, and fatty acid metabolism were completely opposite (Table 2 and Additional file 7: Figure S3).

### Potential function of DELs

**Fig. 3:**
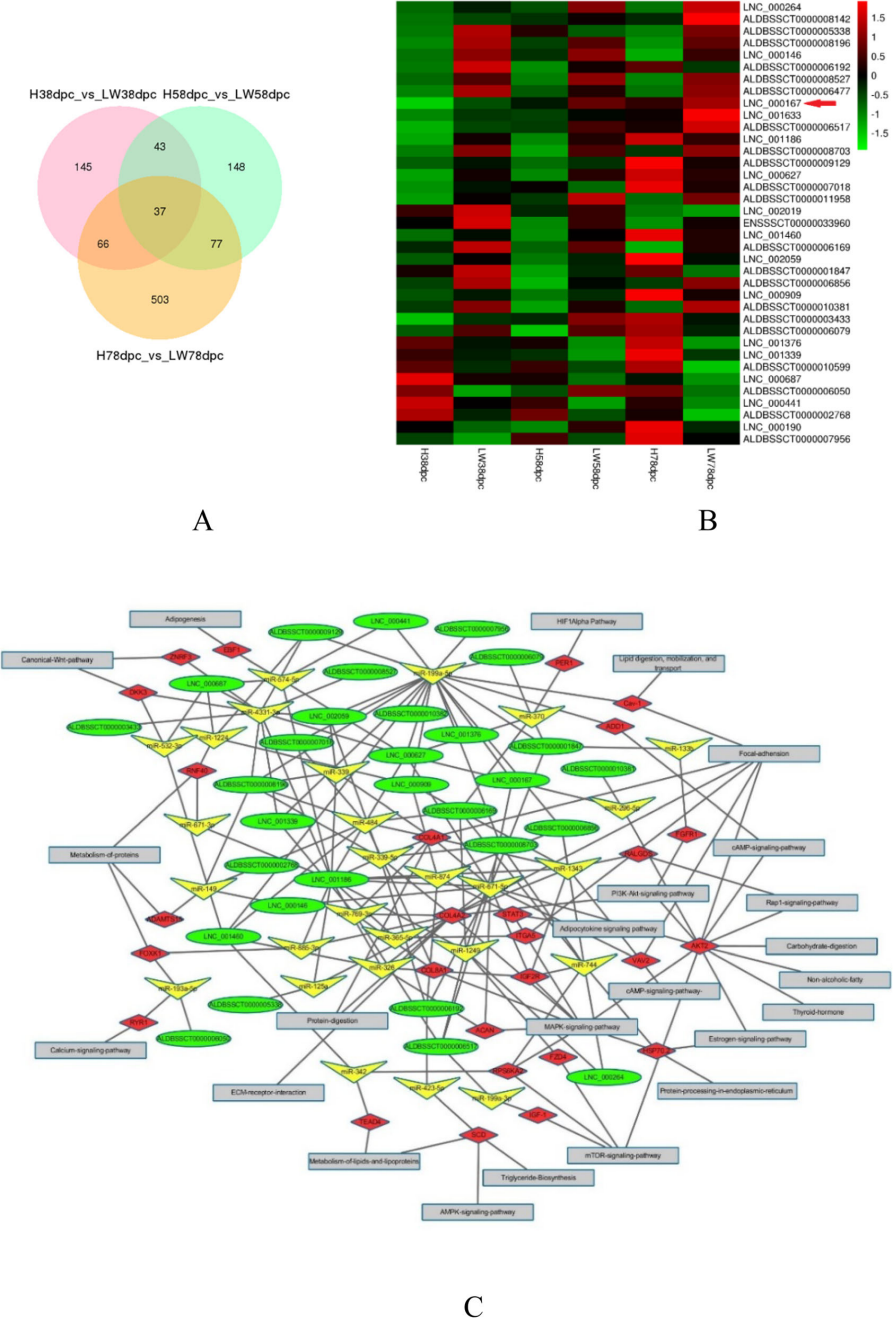
The function of 37 shared DELs between HN and LW at each stage. **a** Venn diagram showing the number of overlapping DELs between HN and LW at three stages. **b** The heatmap of the 37 shared DELs’ expressional level in three stages between HN and LW pigs. Red arrow showed the selected LNC_000167 (IMFlnc1). **c** The ceRNA network of 37 shared DELs. Circular nodes represent lncRNAs; triangular nodes represent miRNAs; diamond nodes represent mRNAs; and square nodes represent pathways

**Fig. 4:**
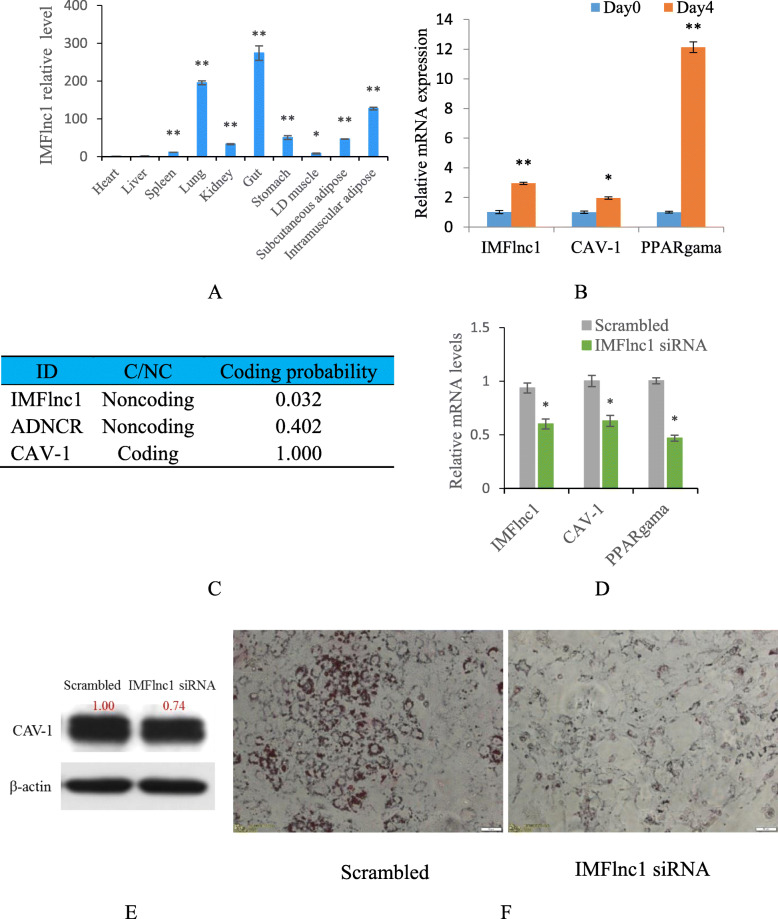
IMFlnc1 was associated with adipogenesis. **a** The expression profile of IMFlnc1 in different tissues by qRT-PCR. **b** The expression level of IMFlnc1 and PPARgama during porcine intramuscular preadipocyte differentiation by qRT-PCR. **c** The coding probability of IMFlnc1, ADNCR and CAV-1 were analyzed by Coding Potential Calculator 2 (CPC2) program. **d** IMFlnc1 siRNA could downregulate expression of IMFlnc1, CAV-1 and PPARgama by qRT-PCR. **e** The effect of IMFlnc1 siRNA on CAV-1 protein expression by Western blot. **f** Inhibition of IMFlnc1 inhibited adipogenesis by Oil Red O staining

The expressional level of 37 shared DELs between these two breeds at three stages are shown in Fig. 3a and b. The ceRNA regulatory network of these shared DELs was constructed and visualized using Cytoscape software, including 30 lncRNAs, 27 miRNAs, 27 mRNAs, and 24 pathways (Fig. 3c). LncRNAs had up to 7 interacting miRNAs, such as ALDBSSCT0000006192, and miR-199a-5p had the most target lncRNAs, seventeen target lncRNAs for each miRNA. Considering the abundance and transcript length, IMFlnc1 was selected for subsequent verification among miR-199a-5p target lncRNAs. IMFlnc1 is located on porcine chromosome 7, and includes two exons. The IMFlnc1 expressional level in intramuscular adipose tissue was higher than that in LD muscle tissue (Fig. 4a), so it was speculated that IMFlnc1 might play an important role in intramuscular adipose tissue, so we firstly verified its regulation role in adipogenesis of porcine intramuscular adipocytes.

### IMFlnc1 promotes adipogenesis of intramuscular adipocytes

RT-qPCR showed that IMFlnc1 showed the highest expressional level in the gut and lungs, followed by in intermuscular fat. Its expression level in subcutaneous fat was lower than it was in intermuscular fat, but higher than it was in LD muscle (Fig. 4a). Moreover, time-course analysis showed that IMFlnc1, CAV-1 and PPARgama were upregulated during the differentiation of porcine intramuscular preadipocyte, which was similar with PPARgama expressional trends (Fig. 4b).

Analyzed by CPC software, IMFlnc1 showed very low coding potential, similar to a well-known lncRNA—ADNCR [22], (Fig. 4c). To explore the function of IMFlnc1 in adipogenesis, we performed knockdown IMFlnc1 in intramuscular adipocytes by lncRNA smart silencer, its RNA level was significantly reduced, and CAV-1 and PPARgama (adipogenic markers) significantly downregulated (Fig. 4d). The downregulation of CAV-1 by IMFlnc1 siRNA was confirmed by Western blot (Fig. 4e). Oil Red O staining indicated that adipogenesis was inhibited by knockdown of IMFlnc1 (Fig. 4f).

### IMFlnc1 and CAV-1 were miR-199a-5p’s target genes

**Fig. 5:**
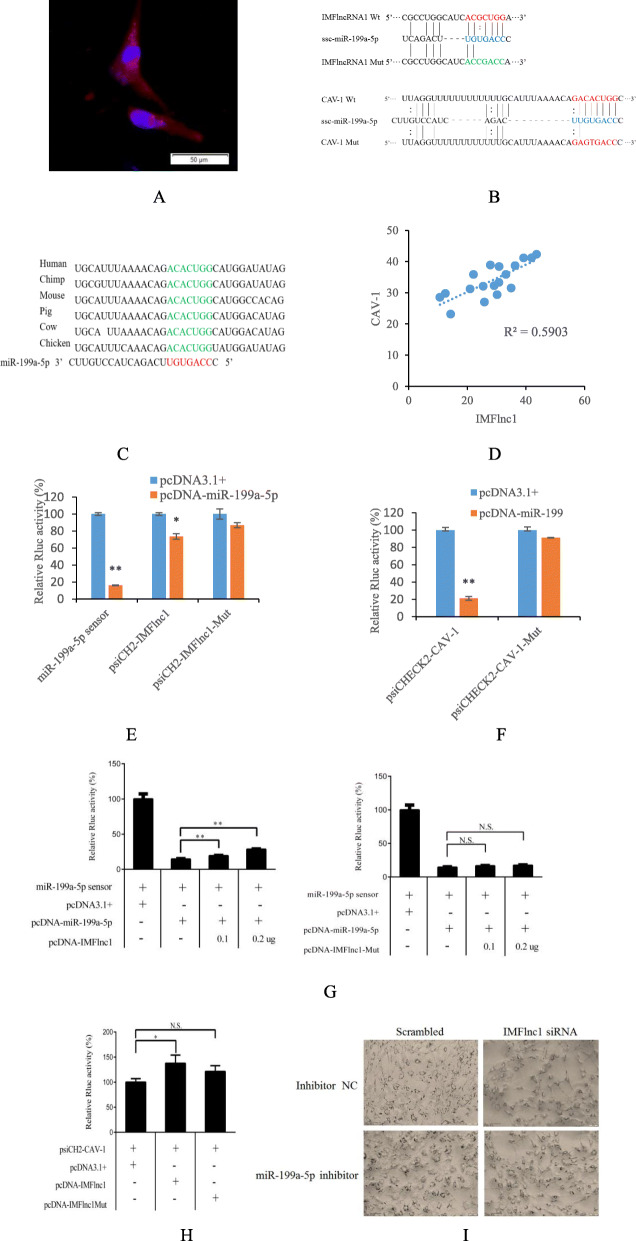
IMFlnc1 regulates CAV-1 expression by sponging miR-199a-5p. **a** Detection of IMFlnc1 in porcine intramuscular adipocyte by RNA fluorescence in situ hybridization (RNA-FISH). Red represents FISH probes of IMFlnc1. Nuclei are counterstained with DAPI (blue). Scale bar, 50 μm. **b** The schematic diagram shows the sequences of IMFlnc1 and CAV-1 with miR-199a-5p, inculding wild-type (Wt) and mutant type (Mut). **c** The binding site of miR-199a-5p at CAV-1 3’UTR (green) are evolutionarily conserved across species. **d** The correlation analysis of IMFlnc1 expression level and CAV-1 mRNA level in 18 porcine LD muscle tissues. **e** IMFlnc1 acted as the target of miR-199a-5p. **f** CAV-1 acted as the target of miR-199a-5p. **g** IMFlnc1 acted as the sponge of miR-199a-5p. IMFlnc1 increases CAV-1 expression (**h**) and adipogenesis (**i**) in a miR-199a-5p dependent manner

IMFlnc1 is mainly localized in preadipocyte cytoplasm (Fig. 5a), so it might participate in the regulation of adipogenesis through ceRNA mechanism. The IMFlnc1-miR-199a-5p-CAV-1 pathway was selected from the ceRNA network to verify its function in adipogenesis. Bioinformatics analysis of the RNAhybrid showed that there exists a binding site of miR-199a-5p in IMFlnc1 and CAV-1 (Fig. 5b), and the binding site in CAV-1 is conservative in different animals (Fig. 5c). The results of RT-qPCR showed that the expressional trends of IMFlnc1 and CAV-1 has a positive correlation in porcine LD muscle (Fig. 5d, *R*^*2*^ = 0.590).

Luciferase assay indicated that miR-199a-5p significantly reduced the fluorescence activity of the sensor and psiCHECK2-IMFlnc1 (*p* < 0.01, Fig. 5e). However, miR-199a-5p couldn’t reduce the fluorescence activity of psiCHECK2-IMFlnc1-Mut, which indicated that miR-199a-5p target IMFlnc1. Similarly, miR-199a-5p also target CAV-1 (Fig. 5f).

### IMFlnc1 participates in adipogenesis by increasing CAV-1

To verify whether IMFlnc1 might sponge miR-199a-5p, the miR-199a-5p sensor was transfected with pcDNA3.1+, pcDNA-miR-199a-5p or pcDNA-IMFlnc1. The pcDNA-miR-199a-5p significantly reduced the fluorescence activity of the miR-199a-5p sensor (*p* < 0.01), but the fluorescence activity could be recovered by IMFlnc1 in a dose-dependent manner (*p* < 0.01, Fig. 5g). However, pcDNA-IMFlnc1-Mut (the binding site of miR-199a-5p was mutated) couldn’t recover the fluorescence activity. It was indicated that IMFlnc1 could sponge miR-199a-5p.

From Fig. 4d and e, mRNA and protein of CAV-1 was downregulated during the reduction of IMFlnc1. To further determine whether IMFlnc1 regulated CAV-1 through miR-199a-5p, psiCH2-CAV-1 was co-transfected with pcDNA3.1, pcDNA-IMFlnc1 or pcDNA-IMFlnc1Mut, respectively. The Rluc activity of CAV-1 was improved by the overexpression of IMFlnc1, but the overexpression of IMFlnc1 with the mutated miR-199a-5p binding sites no longer elicited similar effect (Fig. 5h). And Oil Red O staining confirming that, compared with inhibition of IMFlnc1, the number of mature adipocyte was increased after inhibition of miR-199a-5p and IMFlnc1 (Fig. 5i). In summary, these results indicated that IMFlnc1 might promote adipogenesis by sponging miR-199a-5p.

## Discussion

Previous studies showed that muscle development during the embryonic stages is different between lean- and fat-type pigs. The LD muscle morphological differences between LT and LR pigs were compared by histological section, primary fibers could be found at 35 dpc in LT pigs, but in LR pigs primary fibers could be found at 49 dpc. The secondary fibers formed at 63 dpc in both breeds, LR pigs had more fibers and larger muscle fiber diameter at 91 dpc [23]. Zhao et al., compared muscle morphological differences between TC and Yorkshire (YK) pigs, TC pigs had more myoblasts than YK pigs at 30 dpc, primary fibers could be found at 40 dpc in both breeds, YK pigs had the secondary fibers at 55 dpc but later in TC pigs [12]. These studies indicated that myoblast differentiation, the formation of fibers, and muscle fiber diameter was different between these two type pigs. To study the function of lncRNA in meat quality between the fat- and lean-type pigs, DELs in LD muscle between HN and LW pigs at 38, 58, and 78 dpc were identified. Results indicated that there were more DELs in 58 vs 38 dpc than in 78 vs 58 dpc in HN pigs. However, there was the completely opposite trend in LW pigs. These coincided with the difference in muscle fibers development between fat- and lean-type pigs.

GO analysis of DEL-co-expressed genes was performed to further understand their biological functions (Table 1), and it was indicated that DELs associated with muscle development were continuously upregulated in both breeds, and compared with LW pigs, HN pigs with higher muscle development at 58 dpc, but LW pigs with higher development at 78 dpc. Muscle cell differentiation was continuously upregulated in HN pigs, but only upregulated at 78 dpc in LW pigs. And HN pigs showed a higher differentiation at 58 dpc, but LW pigs showed a higher differentiation at 78 dpc. Similarly, contractile fiber and myofibril were continuously upregulated in both breeds, but at 58 dpc their expression was higher in HN than in LW pigs. Genes in the myofibril assembly pathway were continuously upregulated in HN pigs, but only upregulated at 78 dpc in LW pigs, and at 78 dpc its expressional level was higher in LW pigs. These results indicated that compared with LW pigs, HN pigs showed earlier myogenic differentiation. Muscle development-related biological activities were most active at 58 dpc in HN pigs, but most active at 78 dpc in LW pigs.

KEGG analysis of DEL-co-expressed genes indicated that (Table 2), HIF-1α (hypoxia inducible factor-1α), a key pathway regulating myogenic differentiation, showed higher expression at 58 dpc, but lower expression at 78 dpc in HN than in LW pigs. The pathways associated with energy metabolism showed the same trends, such as biosynthesis of amino acids, adipocytokine pathway, fatty acid metabolism, and fructose and mannose metabolism. These results verified that myogenic differentiation in HN pigs was most active at 58 dpc, so more energy was needed at this period. In contrast, LW pigs showed the most active myogenic differentiation at 78 dpc, so energy-related genes had higher expression at 78 dpc.

Previous researchers found that TC pigs had longer myoblast proliferation phases than WZS pigs [8], and it was predicted that cell proliferation may associated with the differences in muscle mass and body size between these two breeds [10]. In the current study, GO analysis showed that LW had higher cell proliferation than HN pigs at 78 dpc. The insulin [24] and the MAPK [25] pathways both promoted muscle cell proliferation. The insulin pathway showed a lower expressional level in HN than that in LW pigs in all three stages. MAPK showed a lower level in HN than in LW pigs at 58 and 78 dpc. These results verified that myoblast proliferation was lower in HN than in LW pigs, which coincided with more muscle mass in LW pigs.

It was reported that phosphatidylinositol-3-kinases/protein-serine-threonine kinase (PI3K/Akt) participates in mesenchymal stem cells’ adipogenic differentiation [26]. G protein-coupled receptor (GPR) 39 activated the proliferation and differentiation of porcine intramuscular preadipocytes through the PI3K/Akt cell signaling pathway [27]. In this research, PI3K/Akt with a higher level in HN pigs at 78 dpc. The cAMP pathway, which could inhibit adipocyte differentiation and promote lipolysis [28], was continuously downregulated at the prenatal period in both breeds, but its expressional level was lower in HN than in LW pigs in all three stages. These results coincided with the higher IMF content in HN pigs.

The GO and KEGG results of DELs were consistent with previous studies on the differences in embryonic muscle development between fat- and lean-type pigs. To explore how these DELs regulate muscle development, miRanda software was used to analyze the interactions between the 37 shared DELs and miRNAs. As shown in Fig. 4c, many miRNAs enriched in the ceRNA network were related to meat quality. The expression of miR-125, miR-133, and miR-199 in LD muscle was significantly different between German Landrace and Pietrain (Pi) pig breeds at the pre- and post-natal period [29]. Similarly, miR-133 was significantly highly abundant in MS (fat-type) than that in LW (lean-type) pigs at all prenatal stages (35, 55, and 90 dpc) [7]. MiR-199, miR-423, miR-296, miR-193 and miR-125 showed significantly different expressional level between intact male pigs (less fat deposition) and castrated male pigs (more fat deposition) [30]. In obese children, the expressional level of miR-423-5p was increased [31]. MiR-342, miR-193a-3p, miR-365 and miR-125 showed significantly different expressional level between obese and lean individuals [32].

There were also muscle-development-related miRNAs in the ceRNA network, such as miR-133, it showed more highly expressional level in skeletal muscle in MS pigs (fat type) than in LW pigs (lean type) at all prenatal stages [7]. SNPs in miR-133 were significantly associated with loin eye area, total muscle fiber number, and muscle pH [33]. Meanwhile, miR-133 inhibits myoblast proliferation through the ERK (extracellular signal-regulated kinase) signaling pathway [34]. It also participates in the regulation of the metabolic difference between glycolytic and oxidative myofiber [35]. There are also several miRNAs associated with lipid metabolism in the ceRNA network, for example, miR-370 [36], miR-326 [37], miR-574-5p [38], and miR-296 [39].

In the ceRNA network there are the miRNAs associated not only with muscle development, but also with adipocyte development, such as miR-199a-5p, which has the most targets in the current ceRNA network. As the key regulator in the Wnt signaling pathway, miR-199a-5p induced muscle proliferation by targeting *HIF-1* [40]. It also regulated cardiomyocyte apoptosis by targeting *JunB* (JunB proto-oncogene) [41]. In diabetic individuals, miR-199a-5p may be involved in skeletal muscle insulin resistance by inhibiting glucose transporter 4 (*GLUT4*) and hexokinase 2 (*HK2*) [42]. MiR-199a-5p also participates in lipid metabolism, in Italian Large White pigs, miR-199a-5p showed a higher expressional level in the backfat of the lean groups than that in the fat group [43]. Its expressional level was also higher in undifferentiated 3 T3-L1 adipocytes, and rapidly reduced during differentiation [44]. In human bone-marrow-derived mesenchymal stem cells, overexpression of miR-199a-5p inhibited the marker gene of adipocytes-*FABP4* (*ap2*) [45]. In mice, groin fat pads weight was reduced when the *Dnm3os* (DNM3 opposite strand/antisense RNA) was knocked down, which served as a precursor of miR-199a-3p and miR-199a-5p [46]. It was demonstrated that miR-199a-5p regulating retrograde transport and endosome trafficking [47]. And miR-199a-5p participated in the regulation of endocytic transport by controlling important mediator of endocytosis, such as *CAV-1* [48]. In porcine preadipocyte, miR-199a-5p significantly promoted proliferation and reduced adipogenesis by targeting *CAV-1* [49]*.* MiR-199a-5p regulated adipogenic differentiation [50]. In general, miR-199a-5p was not only associated with muscle cells proliferation and differentiation, but also with the pre-adipocytes proliferation and adipogenesis. Similarly, miR-370 was not only related to lipid metabolic homeostasis and lipid deposition [36], but also regulates muscle cell apoptosis [51].

There are several coding genes associated with meat quality, for example adipocyte determination and differentiation dependent factor 1 (*ADD1*) [52], stearoyl-CoA desaturase (*SCD*) [53], *IGF-I* [54]. *CAV-1*, an important fatty acid binding protein, moved from the plasma membrane to lipid droplets under the action of free fatty acids [55]. Compared with Large YK pigs, *CAV-1* showed a significantly higher expressional level in the backfat (*p* < 0.01) and longissimus dorsi muscle (*p* < 0.05) in LT [56]. *CAV-1*-deficient mice showed a lean body phenotype, insulin resistance, and hypertriglyceridemia with adipocyte abnormalities [57]. Activated by TNF-α, *CAV-1* reduced 3 T3-L1 cell differentiation and blocked insulin-mediated glucose uptake [58]. *CAV-1* was associated with lipoprotein metabolism, lipolysis [59], cholesterol homeostasis [60], fatty acid intake [61], and atherosclerosis [62], so *CAV-1* played key regulation role in adipogenic differentiation and glycolipid metabolism.

These lipid metabolism and muscle development related coding genes and miRNAs in the ceRNA network indicated that these DELs may participate in the regulation of meat quality via the ceRNA mechanism. So, the miR-199a-5p-IMFlnc1-*CAV-1* pathway was selected to be verified in porcine intramuscular adipocytes (Figs. 4 and 5). The results indicated that IMFlnc1 had broad-spectrum expression in different tissue types, and its expressional level was upregulated during intramuscular preadipocyte differentiation. The results of Oil red O staining indicated that the reduction of IMFlnc1 inhibits adipogenesis. Luciferase assay revealed that IMFlnc1 could sponge miR-199a-5p, thereby reducing the suppression of miR-199a-5p in *CAV-1*. At the same time, miR-199a-5p also regulates myoblast differentiation. The role of IMFlnc1 in muscle cell proliferation and differentiation would be verified in further research. These findings were of great significance for understanding the molecular regulation mechanisms of meat quality trait differences between fat- and lean-type pigs.

## Conclusions

The expression profiling of lncRNAs in porcine LD muscle at 38, 58, and 78 dpc between HN and LW pigs was investigated. These findings were of great significance for understanding the differences in meat quality trait between fat- and lean-type pigs.

## Methods

### Animals and tissue collection

Five HN and five LW sows (in their second or third parity) were artificially inseminated by using semen from the boars of the same breed (with the same genetic background). One sow from each breed was killed by intravenous injection of sodium pentobarbital (50 mg/kg body weight) at 38, 58, or 78 dpc. Three male and three female fetuses were immediately picked out from the uteri. For 38 dpc, all of the fetuses were selected, and their sex was subsequently identified by the SRY gene, and then three male and three female fetuses were selected for sequencing. LD muscle tissue from the same area was obtained and kept in liquid nitrogen. All pigs were obtained and raised under the same conditions [63] at Henan Xing Rui Agricultural and Animal Husbandry Technology Co., Ltd., (Henan province, China).

### RNA isolation, library preparation, and sequencing

TRIzol reagent (Invitrogen, USA) was used to extract total RNA from 36 LD muscle tissue. The purity, quality, and integrity of the isolated RNA was detected by Agilent 2100 Bioanalyzer (Agilent Technologies, USA) and NanoDrop spectrophotometer (Nano-Drop Technologies, USA). Samples used for library construction should with high RNA integrity number (RIN) value (larger than eight). Residual genomic DNA was removed by using DNase (QIAGEN, USA), and ribosomal RNA were removed by using Ribo-Zero™ rRNA Removal Kit (Epicentre, USA). For each breed, one male RNA sample and one female RNA sample from the same stage were mixed, so three mixed samples were used for sequencing for each stage. The RNA sequencing library Illumina was construct by using TruSeq™ RNA Sample Prep Kit. The libraries’ quality was analyzed by Agilent 2100 Bioanalyzer (Agilent Technologies, USA). Illumina Hiseq 2500 platform was using to sequence the 18 constructed libraries, and generated 125 bp paired-end reads. The clean data was mapped to the porcine reference genome (Sscrofa 10.2, ftp://ftp.ensembl.org/pub/release-84/fasta/sus_scrofa/dna/) by TopHat2 software [64].

### Identification of lncRNAs and differential expression analysis

The identification of lncRNAs followed these five steps: (1) transcripts with more than one single exon were left; (2) transcripts longer than 200 bp were left; (3) annotated lncRNAs in the database that overlapped with the transcripts in this research were left as annotation lncRNAs for subsequent analysis (Cuffcompare software); (4) transcripts with FPKM > 0.5 were left; (5) the transcripts were analyzed by Coding-Non-Coding-Index (CNCI, score < 0), Coding Potential Calculator (CPC, score < 0), k-mer scheme (PLEK, score < 0), and Pfam (E value < 0.001) software. Transcripts that hadn’t passed any of these software tests won’t be considered as lncRNAs.

### Gene ontology (GO) and Kyoto encyclopedia of genes and genomes (KEGG) analysis

Differentially expressed lncRNAs (*p*_adj_ < 0.05 and |log_2_FoldChange| > 0.3) were identified by the DESeq software package. GO enrichment analysis of DELs-co-expressed mRNAs was performed by GOseq [65]. KEGG analysis was performed using KOBAS [66].

### Validation of gene expression in RNA-seq

Ten lncRNAs were selected from the 37 shared DELs, and their expressional trends at 78 dpc between HN and LW pigs were verified by RT-qPCR. Total RNA was converting to cDNA by using PrimeScript RT reagent Kit with the gDNA Eraser (TaKaRa, China) [67]. Each qPCR reaction including 10 μL 2 × SYBR Premix Ex Taq™ (the SYBR Green PCR kit (TaKaRa, China)), 10 μM forward primers and 10 μM reverse primers (Additional file 4: Table S4), and 20 ng template cDNA. PCR amplification including: a 95 °C for 30 s (initial denaturation step), and 38 cycles of 95 °C for 5 s and 60 °C for 34 s. For each qPCR amplification, a technical triplicate was performed, and the 2^-△△Ct^ method was used to calculate the relative RNA expression values [68].

### LncRNA-miRNA-mRNA ceRNA regulatory network

Interaction between the 37 shared DELs and miRNAs was analyzed by the miRanda software. MiRNAs used in the analysis came from the HN and LW pigs’ LD muscle at 38, 58, and 78 dpc, and these data were not published. The construction of the network included three steps, and the methods were the same as those previously described [21].

### Isolation, differentiation, and culture of porcine intramuscular adipocyte

Porcine intramuscular adipocyte were isolated and cultured from HN pigs’ LD muscle following methods as previously described [69]. Intramuscular adipocytes were cultured in a normal-atmosphere incubator at 37 °C and 5% CO_2_ with the normal medium: DMEM/F12 supplemented with FBS (10%) and antibiotics (100 IU/mL penicillin and 100 μg/mL streptomycin). Until 100% confluence, two days later cells were cultured with a differentiation medium (the “normal medium” supplemented with 5 μg/mL of insulin, 1 mM DEX, and 0.5 mM IBMX). Two days later, the cells were cultured with a maintenance medium (the “normal medium” supplemented with 5 μg/mL of insulin) for 2 days. Then the cells cultured with the “normal medium”.

### RNA FISH (RNA fluorescence in situ hybridization)

IMFlnc1 probes labeled with Cy3 were designed and obtained from RiboBio (China). The subcellular localization of IMFlnc1 was detected by using Fluorescent in situ hybridization kit (RiboBio, China).

### IMFlnc1 knockdown

Interference of IMFlnc1 was conducted using a Ribo™ Smart Silencer (Ribobio, China). The smart silencer (IMFlnc1 siRNA, 25, 50, or 75 nM) or Scrambled was transfected to the primary intramuscular preadipocytes (70–80% confluence) by DharaFect 2 (Dharmacon, USA). The full culture media was changed 12 hours later. The interference efficiency was detected 24 hours after transfection (primers showed in Additional file 4: Table S4). Four days and six days after differentiation, cells were used for Western blot and Oil Red O staining, respectively.

For western blot, cells were harvested by RIPA buffer supplemented with PMSF. The protein from each cell lysate was separated on 12% SDS-PAGE gels. The CAV-1 and actin antibody were purchased from Boster (China) and Abcam (UK), respectively. Signals were visualized by ECL using ChemiDoc XRS+ Imaging system (Bio-Rad, USA).

Porcine intramuscular adipocytes (70–80% confluence) were transfected with IMFlnc1 siRNA and inhibitor NC (Ribobio, China), miR-199a-5p inhibitor (Ribobio, China) and Scrambled, IMFlnc1 siRNA and miR-199a-5p inhibitor, Scrambled and inhibitor NC, respectively. Two days after 100% confluence, cells were cultured with a differentiation medium. Six days after differentiation, cells were used for Oil Red O staining.

### Plasmid construction

Full lengths of IMFlnc1 and 3′ UTR of *CAV-1* were cloned into the psiCHECK-2 vector (Promega, USA). The psiCH2IMFlnc1-Mut and *CAV-1* 3′ UTR without the seed sequence of miR-199a-5p were amplified by overlapping PCR. Sensor for miR-199a-5p was constructed by adding two complementary sequences of miR-199a-5p into psiCHECK-2 vector.

### Luciferase assays

HEK293T cells were cultured in 48-well plates, and plasmids were transfected when the cell reached 70% or 80% confluence. Luciferase activities were measured 48 hours after transfection on a Fluoroskan Ascent FL instrument (Thermo Fisher Scientific, USA) by using the Dual-Luciferase Reporter Assay System (Promega). The relative Rluc activity for each sample was calculated by normalized renilla luciferase activity to firefly luciferase activity.

### Statistical analysis

Results were representing as mean ± SE, and it was considered statistically significant when the *p* value < 0.05.

## Supplementary Information

**Additional file 1 **: **Table S1**. Read mapping to reference porcine genome (susScr3).

**Additional file 2 **: **Table S2**. Information of novel lncRNAs.

**Additional file 3 **: **Table S3**. Information of the differently expressed lncRNAs.

**Additional file 4 **: **Table S4**. The information of the primers used for real-time qPCR.

**Additional file 5 **: **Figure S1**. Correlation analysis of lncRNA expression in different samples.

**Additional file 6 **: **Figure S2**. Correlation analysis of lncRNA expression in different samples.

**Additional file 7 **: **Figure S3**. Validation of expression trends of ten randomly selected lncRNAs (five upregulated and five downregulated at 75 dpc) by qRT-PCR. Note: GAPDH used as reference genes, and fold changes calculated using 2^-△△Ct^ method (mean ± SD, *n* = 3, * *p* < 0.05,** *p* < 0.01).

## Data Availability

The datasets used in current study are available in the manuscript and its additional files, and raw sequence data had been submitted to NCBI Sequence Read Archive (SRP243554).

## Abbreviations

ADD1: Adipocyte determination and differentiation dependent factor 1
cAMP: Cyclic adenosine monophosphate
CAV-1: Caveolin-1
C/EBPα: Ccaat enhancer binding protein-α
ceRNA: Competing endogenous RNA
CNCI: Coding-Non-Coding-Index
CPC: Coding Potential Calculator
DEGs: Differentially expressed genes
DELs: Differentially expressed lncRNAs
Dnm3os: DNM3 opposite strand/antisense RNA
dpc: Days post conception
EBF1: Early B cell factor 1
ERK: Extracellular signal-regulated kinase
FABP4: Fatty acid binding protein 4
FPKM: Fragments per kilobase per million
GLUT4: Glucose transporter 4
GPR G: Protein-coupled receptor
HIF-1α: Hypoxia inducible factor-1α
HK2: Hexokinase 2
HN: Huainan pigs
IGF-I: Insulin-like growth factor I
IMF: Intramuscular-fat
IMFlnc1: Intramuscular fat deposition-associated long noncoding RNA 1
JunB: JunB proto-oncogene
KEGG: Kyoto Encyclopedia of Genes and Genomes
LD: Longissimus dorsi
lincRNAs: Large intergenic noncoding RNAs
lncRNAs: Long non coding RNAs
LR: Landrace
LT: Lantang
LW: Large White
MAPK: Mitogen-activated protein kinase
Mef2dL: Myocyte-specific enhancer factor 2D
MS: Meishan
mTOR: Mammalian target of rapamycin
ORF: Open reading frame
Pi: Pietrain
PI3K/Akt: Phosphatidylinositol-3-kinases/protein-serine-threonine kinase
PRDM16: PR domain-containing 16
RIN: RNA integrity number
RNA FISH: RNA fluorescence in situ hybridization
SCD: Stearoyl-CoA desaturase
TC: Tongcheng
TGFBI: Transforming growth factor beta induced
TNF: Total number of fibers
WNT: Wingless/integrated
WZS: Wuzhishan
YK: Yorkshire
