# Supplementary material for: LncRNA IMFlnc1 promotes porcine intramuscular adipocyte adipogenesis by sponging miR-199a-5p to up-regulate CAV-1
Source: BMC Mol Cell Biol. 2020 Nov 4;21:77. doi: 10.1186/s12860-020-00324-8 (PMC7640402; doi:10.1186/s12860-020-00324-8)
Supplement: Supplementary file 1 — Additional file 1 : Table S1. Read mapping to reference porcine genome (susScr3). [file 12860_2020_324_MOESM1_ESM.docx]

Supplementary Table 1. Summary of reads mapping to the reference porcine genome (susScr3).

| Sample name | Total reads | Total mapped | Multiple mapped | Uniquely mapped | Map to '+' | Map to '-' | Nonsplice reads | Splice reads |
| --- | --- | --- | --- | --- | --- | --- | --- | --- |
| H38dpc1 | 99372344 | 78.50% | 10.30% | 68.21% | 33.99% | 34.22% | 49.04% | 19.17% |
| H38dpc2 | 91621774 | 79.50% | 12.96% | 66.54% | 33.11% | 33.43% | 45.50% | 21.03% |
| H38dpc3 | 99373134 | 78.77% | 9.60% | 69.17% | 34.44% | 34.72% | 47.75% | 21.42% |
| H58dpc1 | 92246214 | 79.67% | 16.20% | 63.47% | 31.56% | 31.91% | 43.67% | 19.80% |
| H58dpc2 | 96108168 | 79.54% | 12.76% | 66.78% | 33.25% | 33.53% | 47.20% | 19.58% |
| H58dpc3 | 90404638 | 78.70% | 14.03% | 64.67% | 32.20% | 32.47% | 46.27% | 18.40% |
| H78dpc1 | 122510774 | 79.66% | 12.70% | 66.96% | 33.46% | 33.50% | 48.98% | 17.98% |
| H78dpc2 | 118192178 | 79.70% | 10.68% | 69.02% | 34.48% | 34.54% | 50.60% | 18.42% |
| H78dpc3 | 121244286 | 79.35% | 11.38% | 67.97% | 33.96% | 34.01% | 50.44% | 17.53% |
| LW38dpc1 | 119571668 | 80.37% | 15.60% | 64.77% | 32.29% | 32.48% | 45.62% | 19.15% |
| LW38dpc2 | 100389578 | 80.85% | 10.73% | 70.12% | 35.00% | 35.12% | 50.59% | 19.53% |
| LW38dpc3 | 112166838 | 80.43% | 10.52% | 69.91% | 34.81% | 35.10% | 49.44% | 20.47% |
| LW58dpc1 | 109820356 | 80.05% | 14.11% | 65.94% | 32.91% | 33.03% | 46.81% | 19.12% |
| LW58dpc2 | 111416898 | 80.58% | 13.15% | 67.43% | 33.66% | 33.76% | 47.74% | 19.68% |
| LW58dpc3 | 121403676 | 80.04% | 12.48% | 67.56% | 33.72% | 33.85% | 48.42% | 19.14% |
| LW78dpc1 | 108421388 | 79.19% | 15.73% | 63.46% | 31.67% | 31.79% | 45.06% | 18.40% |
| LW78dpc2 | 116506256 | 78.92% | 14.32% | 64.60% | 32.23% | 32.38% | 46.70% | 17.90% |
| LW78dpc3 | 110401406 | 79.57% | 14.47% | 65.11% | 32.52% | 32.59% | 45.59% | 19.52% |
