# Supplementary material for: LncRNA IMFlnc1 promotes porcine intramuscular adipocyte adipogenesis by sponging miR-199a-5p to up-regulate CAV-1
Source: BMC Mol Cell Biol. 2020 Nov 4;21:77. doi: 10.1186/s12860-020-00324-8 (PMC7640402; doi:10.1186/s12860-020-00324-8)
Supplement: Supplementary file 4 — Additional file 4 : Table S4. The information of the primers used for real-time qPCR. [file 12860_2020_324_MOESM4_ESM.docx]

| Name | Sequence (5'-3') | Size (bp) |
| --- | --- | --- |
| LNC_001633 | F: TCTGAAGAACGCCCTACTTATCG | 223 |
|  | R: CTTGGACTTGGTGGCTTTGAGA |  |
| LNC_000167(IMFlnc1) | F: TTCCTTCTGACTCCCGTTCTCC | 245 |
|  | R: CCTGGATTTGGGTTAGTTGTGGT | |
| ALDBSSCT0000008142 | F: GTCAAGGAGACCCAAGAAAGCA | 153 |
|  | R: TGGATGGCTTGAGCTGTTTCC |  |
| ALDBSSCT0000010381 | F: TAAGGCGTGTCAGGAGGAAGGT | 276 |
|  | R: TCGTGCTGTTTGCCAGGGA |  |
| LNC_000687 | F: CCTCCATTCCATGTCCTCAACT | 121 |
|  | R: TGCCCTCTGAACATCCTATAACCT | |
| ALDBSSCT0000010599 | F: TGCATTTAACGGGCACTTCTAA | 156 |
|  | R: CTCACCACATTCTTCCCAGCAC |  |
| ALDBSSCT0000006477 | F: CCTGTTCCAGTTGCCTTGAGA | 179 |
|  | R: CATCCCTGACCCTGCTCCTA |  |
| ALDBSSCT0000003433 | F: TTCCCTGTTGAGTCCTGTTGCT | 229 |
|  | R: AGTTGGCAAGAGCCTACCCTC |  |
| ALDBSSCT0000009129 | F: CTGGAGGTCCTGGAAGTAGCC | 243 |
|  | R: CCTGGGATTGAAAGCCCTGTAT |  |
| LNC_0001376 | F: AAGTGAGGTGGCATCCCTGTAT | 113 |
|  | R: CTTTCCACCTCCAGCCCTTC |  |
| CAV-1 | F: ATCCCAAGCATCTCAACG | 157 |
|  | R: AAGAGGGCAGACAGCAAA |  |
| PPARgama | F: AGGACTACCAAAGTGCCATCAAA | 142 |
|  | R: GAGGCTTTATCCCCACAGACAC |  |
| Gapdh | F: GTGAAGGTCGGAGTGAACGGA | 252 |
|  | R: CCATTTGATGTTGGCGGGAT |  |

Supplementary Table 4. The information of the primers used for real-time qPCR
