# Supplementary figures and images for: LncRNA IMFlnc1 promotes porcine intramuscular adipocyte adipogenesis by sponging miR-199a-5p to up-regulate CAV-1
Source: BMC Mol Cell Biol. 2020 Nov 4;21:77. doi: 10.1186/s12860-020-00324-8 (PMC7640402; doi:10.1186/s12860-020-00324-8)

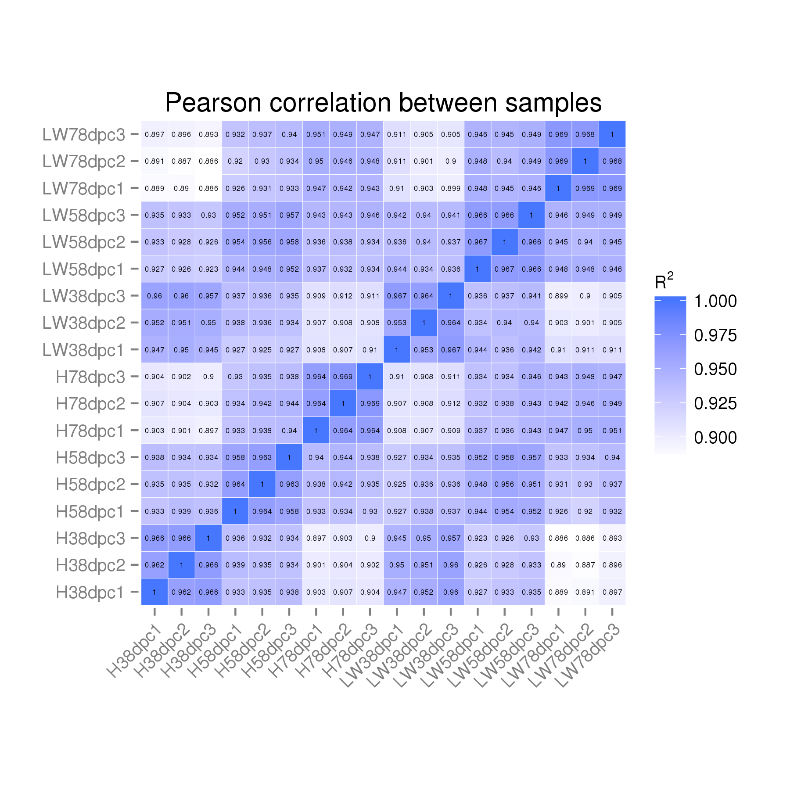


Supplementary Figure1. The correlation analysis of lncRNAs expression in different samples

Supplement: Supplementary file 5 — Additional file 5 : Figure S1. Correlation analysis of lncRNA expression in different samples. [file 12860_2020_324_MOESM5_ESM.docx]

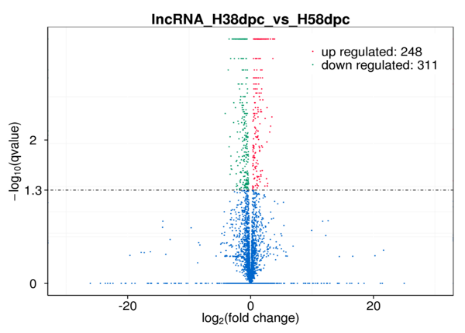

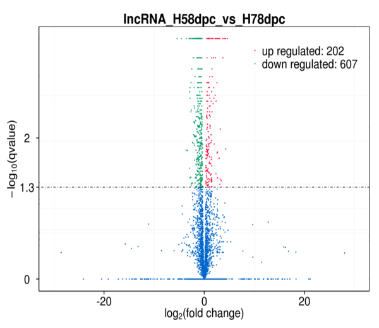

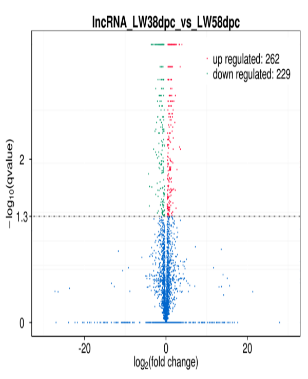

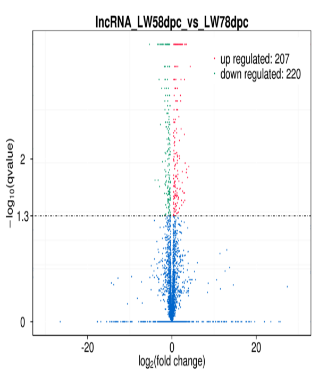

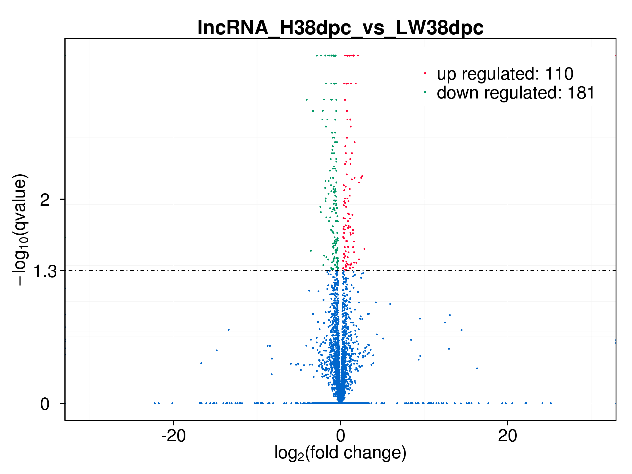

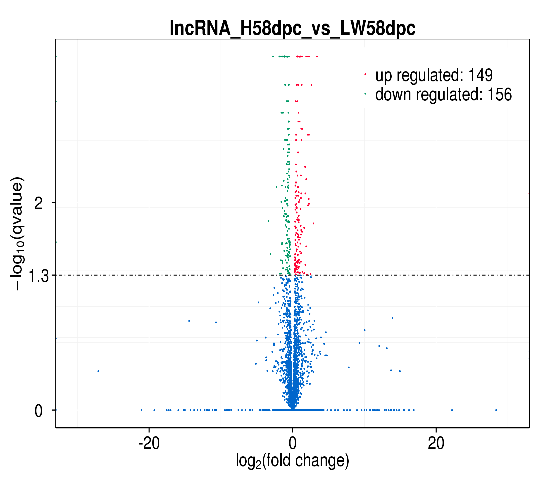

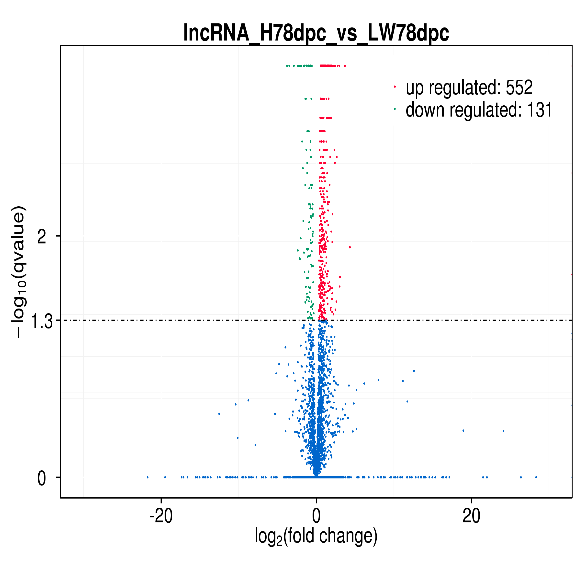


Supplementary figure. 2 The correlation analysis of lncRNAs expression in different samples

Supplement: Supplementary file 6 — Additional file 6 : Figure S2. Correlation analysis of lncRNA expression in different samples. [file 12860_2020_324_MOESM6_ESM.docx]
