# Supplementary material for: LncRNA IMFlnc1 promotes porcine intramuscular adipocyte adipogenesis by sponging miR-199a-5p to up-regulate CAV-1
Source: BMC Mol Cell Biol. 2020 Nov 4;21:77. doi: 10.1186/s12860-020-00324-8 (PMC7640402; doi:10.1186/s12860-020-00324-8)
Supplement: Supplementary file 7 — Additional file 7 : Figure S3. Validation of expression trends of ten randomly selected lncRNAs (five upregulated and five downregulated at 75 dpc) by qRT-PCR. Note: GAPDH used as reference genes, and fold changes calculated using 2-△△Ct method (mean ± SD, n = 3, * p < 0.05,** p < 0.01). [file 12860_2020_324_MOESM7_ESM.docx]

Supplementary Fig.3 Validation the expression trends of the ten randomly selected lncRNAs (five upregulated and five downregulated at 75dpc point) by qRT-PCR.

Note: The GAPDH was used as reference genes, the fold change was calculated using the 2^-△△Ct^ method. (Mean±SD，n=3，*p < 0.05, **p < 0.01).
